# Recent Innovations in Footwear and the Role of Smart Footwear in Healthcare—A Survey

**DOI:** 10.3390/s24134301

**Published:** 2024-07-02

**Authors:** Pradyumna G. Rukmini, Roopa B. Hegde, Bommegowda K. Basavarajappa, Anil Kumar Bhat, Amit N. Pujari, Gaetano D. Gargiulo, Upul Gunawardana, Tony Jan, Ganesh R. Naik

**Affiliations:** 1 Department of Electronics & Communication Engineering, NMAM Institute Technology, NITTE (Deemed to be University), Nitte 574110, India; pradyumna@nitte.edu.in (P.G.R.); roopabhegde@nitte.edu.in (R.B.H.); bgowda_kbl@nitte.edu.in (B.K.B.); anilkumarbhat@nitte.edu.in (A.K.B.); 2 School of Physics, Engineering and Computer Science, University of Hertfordshire, Hertfordshire AL10 9AB, UK; amit.pujari@ieee.org; 3 School of Engineering, University of Aberdeen, Aberdeen AB24 3FX, UK; 4 School of Engineering, Design and Built Environment, Western Sydney University, Penrith, NSW 2751, Australia; g.gargiulo@westernsydney.edu.au (G.D.G.); u.gunawardana@westernsydney.edu.au (U.G.); 5 The MARCS Institute for Brain, Behaviour, and Development, Western Sydney University, Penrith, NSW 2751, Australia; 6 Translational Health Research Institute, Western Sydney University, Penrith, NSW 2751, Australia; 7 The Ingham Institute for Applied Medical Research, Liverpool, NSW 2170, Australia; 8 Centre for Artificial Intelligence Research and Optimization (AIRO), Design and Creative Technology Vertical, Torrens University, Ultimo, NSW 2007, Australia; tony.jan@torrens.edu.au; 9 College of Medicine and Public Health, Flinders University, Adelaide, SA 5042, Australia; 10 Design and Creative Technology Vertical, Torrens University, Wakefield Street, Adelaide, SA 5000, Australia

**Keywords:** assistive technology, deep learning, diabetes management, energy harvesting, health monitoring, smart footwear, technological advancements in footwear, IoT

## Abstract

Smart shoes have ushered in a new era of personalised health monitoring and assistive technologies. Smart shoes leverage technologies such as Bluetooth for data collection and wireless transmission, and incorporate features such as GPS tracking, obstacle detection, and fitness tracking. As the 2010s unfolded, the smart shoe landscape diversified and advanced rapidly, driven by sensor technology enhancements and smartphones’ ubiquity. Shoes have begun incorporating accelerometers, gyroscopes, and pressure sensors, significantly improving the accuracy of data collection and enabling functionalities such as gait analysis. The healthcare sector has recognised the potential of smart shoes, leading to innovations such as shoes designed to monitor diabetic foot ulcers, track rehabilitation progress, and detect falls among older people, thus expanding their application beyond fitness into medical monitoring. This article provides an overview of the current state of smart shoe technology, highlighting the integration of advanced sensors for health monitoring, energy harvesting, assistive features for the visually impaired, and deep learning for data analysis. This study discusses the potential of smart footwear in medical applications, particularly for patients with diabetes, and the ongoing research in this field. Current footwear challenges are also discussed, including complex construction, poor fit, comfort, and high cost.

## 1. Introduction

In recent years, technological advances in the Internet of Things (IoT) and wearable devices have penetrated the shoe industry, driving smart shoe design. Integrating electronic and mechanical components has enabled the evolution of the shoe into a smart shoe. Smart footwear (SF) uses personalised cell phone applications, enabling custom user experience and self-monitoring [1]. Namely, SF can capture, monitor, and record activities of daily living, tracking a range of vital characteristics such as (foot) pressure points, posture correctness, body fatigue level, temperature (within the footwear), number of steps completed, weight of a person, and real-time location. The data can be automatically analysed to aid in diagnosis and/or as personalised feedback to users, measuring real athletic performance, tracking fitness, and evaluating health metrics. The efforts in this area aim to integrate technology to improve comfort, convenience, and good health [2].

This survey highlights how continuous research and technological progress spark the creation and advancement of SF for diverse applications. Driven by this capability, businesses are actively innovating in the SF sector to address the rising demand from consumers. By providing fundamental technological elements of SF and exploring how current technologies can be harnessed to streamline application processes, as well as an outline of future perspectives concerning SF technology, this survey offers a concise overview of the roadmap for integrating modern technologies into footwear for improved lifestyles.

**Figure 1:**
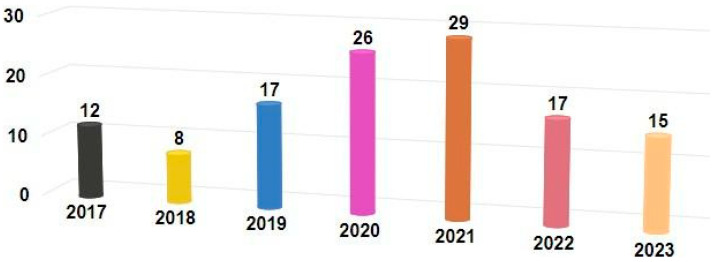
Articles included in the review.

In this study, we searched the Scopus database, PubMed, and IEEE Xplore using the following keywords: “smart footwear”, “IoT footwear”, “intelligent footwear”, “tracking footwear”, “machine learning”, “deep learning”, “real-time monitoring”, and “diabetic footwear.” Aiming to provide recent trends and developments in smart footwear technology, we limited our search to papers published between 2017 and 2023, and only the papers applicable to healthcare were reviewed. The number of published articles included in the review is shown in Figure 1. The number of recent publications on SF reflects increased research and innovation activity in this domain, perhaps motivated by increased consumer/business need for innovative SF.

The present study explores the potential of SF within the healthcare domain. Initially, we emphasise the critical role of sensors in SF functionality. This is followed by a concise overview of current SF implementations and their principal healthcare applications. Subsequently, this paper performs a more in-depth examination of three key functionalities of SF for healthcare purposes: (1) performance tracking, (2) patient monitoring, and (3) detection and recognition. We then provide a brief survey of commercially available smart footwear options. To conclude, the paper synthesises key observations gleaned from the reviewed literature and explores potential future directions for SF research.

## 2. Overview of Smart Footwear—Sensors and Design

SF integrates sensors and innovative designs to enhance functionalities. The present section describes the various design strategies, different types of sensors, and various SF available in the market.

### 2.1. SF Sensors and Design

**Figure 2:**
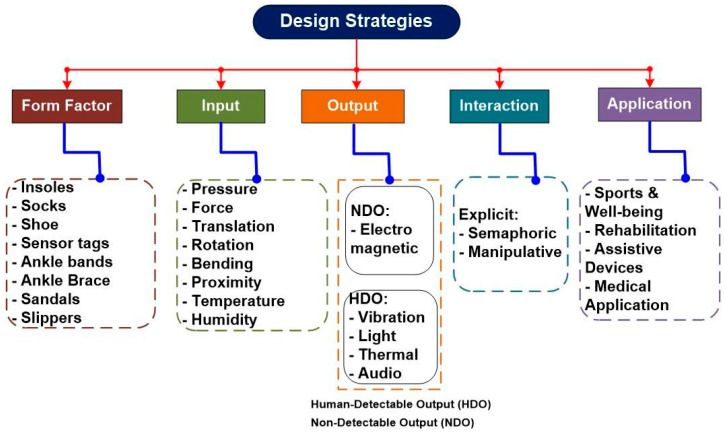
Various design aspects of smart footwear.

SF can be broadly categorised into (a) passive, harvesting the energy required from mechanical (limb) movements, and (b) active, working based on sensors. Notably, the development of modern SF often involves a fusion of both these categories, resulting in hybrid models that uses elements from both passive and active technologies to enhance their overall functionality and performance. Such smart footwear can be used for various applications. The design and development of smart footwear involve multiple aspects, as depicted in Figure 2.

The listed applications are related to human health monitoring. However, SF’s applications are not only limited to human health monitoring but are also used as a tracking and support device. Sensors are electronic devices that, in the SF context, record relevant physical and analogue parameters (such as pressure, temperature, and movement) and convert them into digital form, which can be processed to draw insights into the activities of daily living (e.g., a number of steps, foot pressure points/gait etc.). There are sensors available, and the choice of the sensor depends on the SF requirements. Table 1 lists sensors that are commonly used in SF.

### 2.2. Various Types of SF Available in the Market

Generally, younger generations are more inclined towards technology-incorporated products. Smart footwear is one such product that provides personalised feedback. Perhaps this is one of the reasons that smart footwear expanded at a robust compound annual growth rate (CAGR) of 22.7% during the forecast period 2023–2033. The market is expected to hold a share of USD 269 million in 2023, while it is anticipated to surpass USD 2.1 billion by 2033 [13]. 

**Figure 3:**
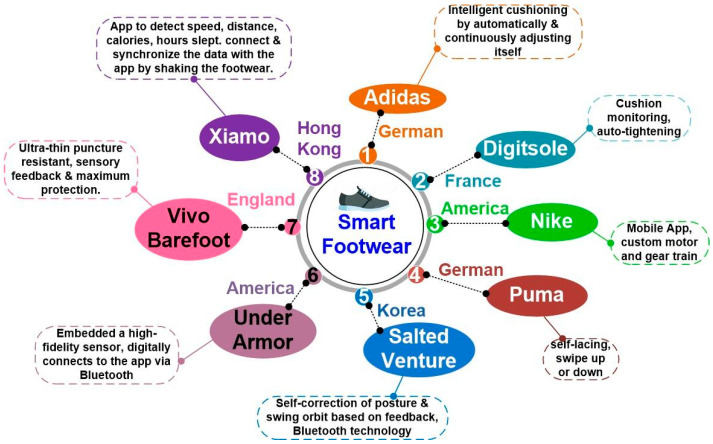
Smart footwear companies on the global market.

**Figure 4:**
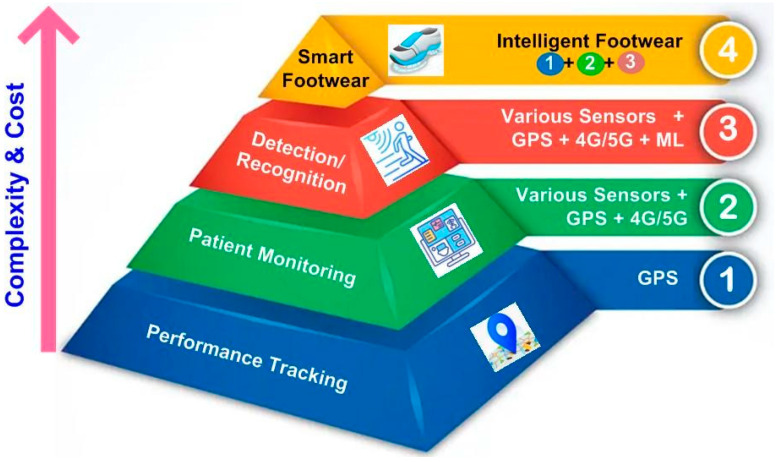
Hierarchy for selection of footwear in healthcare.

The current section attempts to provide details on the available smart footwear in the market. Figure 3 depicts the smart footwear companies in the global market. The figure offers popular manufacturers of smart footwear and their functions. The key feature for market growth is increasingly high expenditure on footwear by consumers. Companies are competing to meet the growing demands of consumers. However, the design complexity and cost appear to be proportional to the features of the SF, as shown in Figure 4.

It is evident from the figure that the computational complexity increases with an increase in the functions. This leads to hardware and software complexity, resulting in increased cost. However, the trend towards SF is growing yearly, so manufacturers are continuously upgrading the technology to meet the growing demand. The details of the SF available in the market are provided in Table 2.

## 3. Smart Footwear Applications

**Figure 5:**
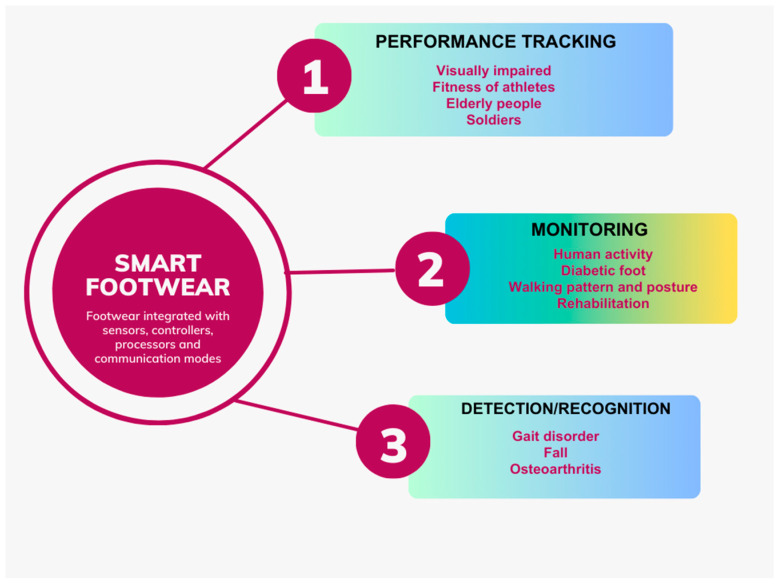
Main applications of smart footwear.

In recent years, the utility of SF has been commonly found in performance tracking, health monitoring, and the detection of specific disorders, as summarised in Figure 5. This section provides an overview of the state-of-the-art methods used in these applications.

**Figure 6:**
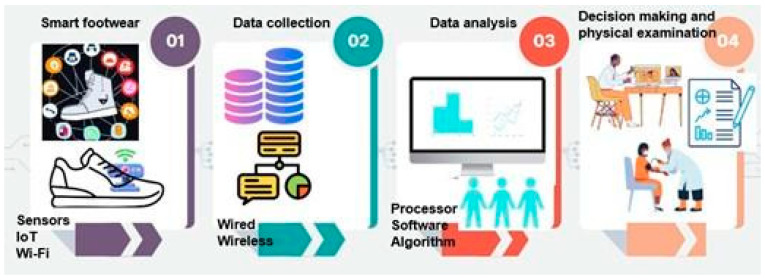
Generic workflow of IoT-based smart footwear.

Once the SF is designed to respond to a specific user need, a specific sensor is selected and integrated into the SF. The data acquired are used to perform decision-making and provide feedback to users. Figure 6 visually illustrates this process. For example, common in SF is an inbuilt observing circuit that gives footstep count, weight distribution, walking speed, travel mapping/distance, and pro-health tips [15]. Once the sensor collects the data, these data are sent to the processing system for data analysis. This requires controller boards typically employing low-power microcontrollers such as the ones powering Arduino, Raspberry Pi, etc. Wireless communication is commonly used over wired communications to transfer data. Automated data analysis and results can be displayed in SF, connected to a dedicated app [15].

Finally, a decision is made based on the analysis reports, which can be generated automatically. Analysis reports can enable remote assistance. Considering the implications for a person’s health and SF’s input in aiding medical decision-making, SF needs to be clinically validated, robust, safe, and in line with the latest patient (personal) data privacy recommendations.

### 3.1. Application 1: Performance Tracking

This section focuses on the state-of-the-art methods reported for tracking the visually impaired, measuring athlete performance, and monitoring older adults and soldiers. Globally, at least 2.2 billion people have some form of vision impairment, including blindness [16]. Visually impaired individuals find it difficult to navigate independently from one place to another. SF can assist the visually impaired in safely navigating. The use of ultrasonic sensors with an Arduino UNO [17,18,19,20], Node MCU [21], Raspberry Pi [22], and microcontroller [23,24] can be found in the SF literature. This footwear can detect obstacles and guide the user to avoid them. Integrating GPS tracking with wireless charging systems, as proposed by Thanuja et al. [22], can provide real-time location information about the user, allowing their friends or family members to be informed when needed.

An extensive study conducted by Hersh et al. [25] discussed various types of wearable devices designed for the travelling aid of the visually impaired. They [25] described SF with ultrasonic obstacle detection and additional functions such as a water detection sensor to detect wet floors, a 3-axis accelerometer, and a 3-axis gyroscope for falls. However, this review focused mainly on design issues. Another study observed secure movement with low faulty errors when the visually impaired were tested with smart footwear with obstacle detection, wet sensing, and fall detection functions [26]. In addition, smart footwear was designed to help blind people find their way back to their destination in case they miss their route [27].

In addition to tracking, physical parameters such as speed, pressure, and acceleration can be measured with sensors integrated into footwear. Also, physiological parameters such as oxygen saturation level, body temperature, and blood pressure can be measured. This would help in understanding the training level and performance of athletes. An investigation by Knopp et al. [28] revealed that the running economy could be improved by runners using smart footwear. A recent study by Gulas and Imre [29] mentioned that along with improving the technological aspects, it is necessary to look into the textile quality and material used for developing smart footwear. Another study reported the importance of smart socks and in-shoe systems for sports and medical applications [30]. Sports injury is an unavoidable circumstance, and this requires rehabilitation for a speedy recovery. Also, gait analysis may be able to detect any severe injury. A study by Lianzhen and Hua [31] evaluated athletes’ performance rehabilitation by providing Internet of Health Things (IoHT) guidance.

Furthermore, a porous polymer dielectric was used for capacitive sensors to improve the pressure sensing of capacitive sensors for measuring heel pressure while running and walking for athletes [32], yielding considerably greater signal changes than the other dielectric materials. Barratt et al. [33] tested the reliability of plantar pressure measurement. They validated it by comparing the performance of the smart insole with PedarX and concluded that a wireless insole might be more practical than a wired system. An article by Beckham [34] described next-generation athlete shoes that allow users to customize the look, fit, and responsiveness of kicks. Also, such shoes can transmit data to the cloud and will receive feedback to fine-tune the workout by connecting to Facebook or X (formerly Twitter). Though there is a need for automated continuous monitoring and assistance for athletes, another major requirement is monitoring for older adults. Worldwide, the elderly population is growing; according to the WHO, it is likely to reach 2.1 billion by 2050 [35].

With ageing comes the risk of age-related health problems. For example, the WHO reports approximately 684,000 fall-related deaths every year worldwide, which are likely to increase with the ageing population [36]. Smart footwear can help minimise falls by monitoring older adults. An article by Schiltz [37] indicated that smart footwear with GPS devices benefits older adults, especially those with Alzheimer’s or cognitive disorders. Li [36] identified the core risk factors to be addressed while designing footwear for fall detection, and proposed an optimised design. A possible smart footwear design for location tracking and health monitoring of older adults with sensors, GPS, and RFID technology was demonstrated by Cheng et al. [38]. SF can be particularly useful when the user cannot access or support a career. In this direction, smart footwear designs and proposals can be found in the literature [39,40,41,42]. The state-of-the-art methods addressed tracking the elderly and monitoring walking speed [39]. Older adults can be safeguarded either by carers or by using technology-driven systems. Defence systems, including the army, navy, and air force, face the issue of tracking soldiers. Smart footwear is a feasible solution for tracking soldiers. Smart footwear-based remote monitoring and location tracking of soldiers can be found in the literature using LoRa [43] for longer-distance communication and mobile applications [44]. The reported articles addressed location tracking and lack of monitoring of physical or physiological parameters. An extensive 2018 study by Friedl [45] focused on all possible wearable devices, including smart footwear, for monitoring soldiers remotely. This study brought out the challenges and future directions. Both health monitoring and position tracking of soldiers were addressed using various sensors, PIC microcontrollers, and GPS [46]. An additional panic button was provided at the soldiers’ end to ask for help from the base camp. 

**Figure 7:**
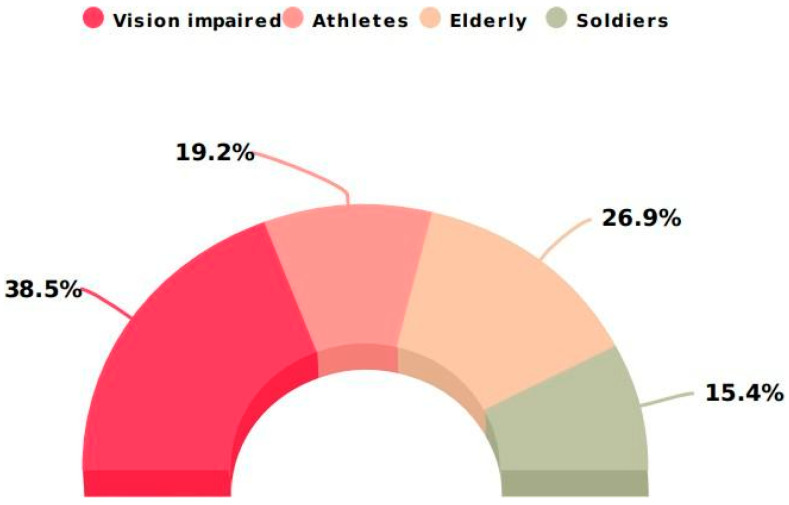
Number of articles addressing performance tracking.

The above section provided an overview of studies addressing performance tracking used in four applications: (a) vision impairment, (b) athletics, (c) the elderly, and (d) defence. Figure 7 depicts the proportion of articles published about each application category.

### 3.2. Application 2: Patient Monitoring

In recent years, wearable technology developments have enabled remote patient monitoring in healthcare centres and homes. Monitoring may include daily activity recognition, monitoring patients with walking issues, or monitoring patients with specific conditions such as diabetes, cardiac issues, gait disorder, etc. Human activity recognition (HAR) is extensively used in various applications such as day-to-day activities, health monitoring, fitness tracking, and monitoring of the physically impaired. Wearable devices available for HAR smart footwear have been gaining popularity in recent years. An investigation by Truong et al. [47] revealed that the wrists or feet are suitable for remote HAR. Hence, footwear is a feasible solution for recording human activity and analysing it remotely. Pressure, inertial, or both sensors can be used for HAR application. A study found that inertial sensors are reliable for recognising dynamic activities, while pressure sensors are reliable for stationary activities [48]. However, the utilization of pressure sensors can also be seen in the literature for recognizing dynamic activities [49,50]. Hence, using both inertial and pressure sensors is suggested for efficient outcomes. Recognition is a task that requires decision-making by processing sensor data. Machine Learning (ML) is identified as most suitable for recognition tasks. The use of Deep Learning (DL) for HAR can be found in the literature [51,52]. Plantar pressure measurement is an important aspect of clinical studies, so it is essential to calibrate the plantar pressure value given by the sensor before integrating it with the footwear. An experimental evaluation by Kakarla [53] disclosed that ML can model smart footwear with the required pressure measurement.

Further, a random forest (RF) algorithm was employed by Ren [50] for daily activity recognition by measuring plantar pressure. Capacitive sensors showed promising results for measuring plantar pressure [54,55], allowing HAR. Another study by Pham et al. trained a DL algorithm using accelerometer data obtained from smart footwear and reported an accuracy of 93% [56]. Image-based 3D analysis was conducted to test the effectiveness of such smart footwear on healthy volunteers [57]. Modern smartphones already come with an array of inbuilt sensors, and a study by Dogan et al. [58] demonstrated the use of smartphone sensors for HAR by employing DL. A daily activity recognition system can be used to monitor healthy people. Disease-specific monitoring, however, requires several parameters specific to the disorder. Hence, SF specific to particular conditions may involve additional hardware and software tools.

**Figure 8:**
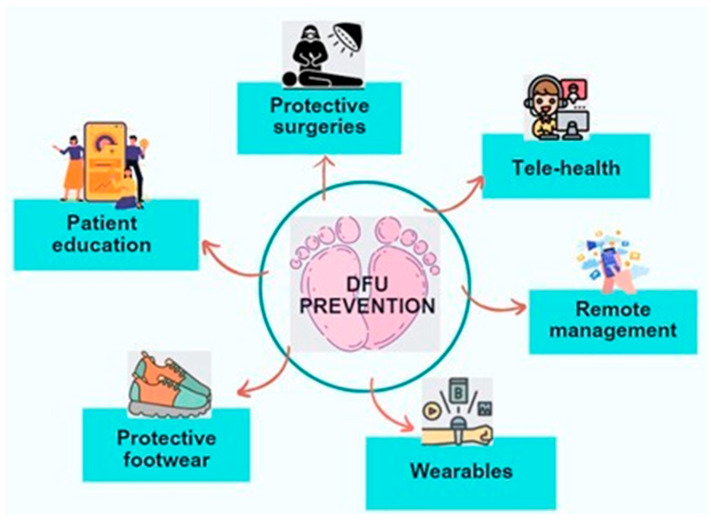
Preventive actions for DFU.

One such widespread condition that requires regular monitoring is diabetes. Diabetes is a chronic disease that damages the heart, blood vessels, eyes, kidneys, and nerves. About 422 million people worldwide have diabetes, the majority living in low- and middle-income countries, and 1.5 million deaths are directly attributed to diabetes each year [59]. Within the diabetic population, the diabetic foot ulcer (DFU) is a life-threatening complication. A recent study reported that the mortality rate due to DFUs was high and was approximately 50% in five years [60]. Hence therapeutic footwear is commonly recommended for early detection and diagnosis. However, this requires continuous monitoring of such patients. Various precursors and risk factors of DFUs include joint contractures, Arthritis, and callus formation. A pronged approach that includes patient education, appropriate footwear selection, telehealth, and proactive surgical interventions is essential to prevent new and recurrent DFUs [61], as depicted in Figure 8. Smart footwear is a feasible solution to monitor patients remotely, helping to manage DFUs more effectively.

DFUs are a common complication of diabetes, often resulting from nerve damage or poor blood circulation. Hence, managing DFUs is crucial due to their potential to lead to severe infections. Smart footwear, integrated with advanced technologies, has emerged as a promising tool in the early detection, monitoring, and managing of diabetic foot complications. Hence, several innovative approaches are emerging to monitor DFUs by integrating sensors in footwear. Moulaei et al. [62] and Altaf et al. [63] showed accurate measurements of pressure, humidity, and temperature of patients’ feet, and sent these data to their smartphones by Bluetooth modules. A similar study by Sousa et al. [64] developed a need-based SF to monitor plantar pressure. A mobile-based plug-and-play device (“Dia Shoe”) developed by Kularathne et al. [65] efficiently measured the temperature, humidity, weight, and step count of the patient through a mobile application. A prototype SF model integrated with foot pressure and blood flow monitoring systems with wireless data transmission [66] showed promising results for plantar pressure measurement [66]. A proof-of-concept study [67] paired a smartwatch and SF consisting of eight pressure sensors for monitoring plantar pressure and alerting the individual through the watch. A flexible printed circuit board (PCB)-based insole design [68] incorporated eight capacitive sensors sending data to a personal computer (PC) using a Bluetooth module, enabling continuous monitoring of plantar pressure for real-time data analysis and evaluation. A study [69] recorded data associated with patients’ preference for footwear, insole design, and quality-of-life-related information for further analysis and declared that patient-centric SF design is the key point for therapeutic outcomes. A feasibility study [70] for measuring plantar pressure revealed that insole optimisation holds promise for evaluating the effectiveness of SF in monitoring diabetic neuropathy. The temperature of the skin surface varies in the presence of an ulcer or wound on the surface. This fact was utilized [71,72] to design SF integrating footwear and temperature sensors for early detection of foot ulcers in diabetic patients. Additionally, Zhang et al. studied deformation in the foot due to diabetes, specifically in women, by measuring plantar pressure [73].

Designing smart footwear involves several features, such as the selection of sensors, number of sensors, placement of sensors, communication mode, selection of controllers and processors, etc. One study [72] provides a detailed description of the design aspects of smart footwear. However, the choice of textile materials used in footwear can alter its efficiency [74,75]. Smart footwear must satisfy user needs and meet aesthetic/perception thresholds [76]. Sometimes, older adults may be hesitant to appreciate and accept smart/modern technology. This might cause mental disturbance during the usage of such systems. However, a study by McDonald has shown that ML models can be used to monitor psychosocial factors and indicate a preference for using smart footwear and adapting to the technology [77]. Designing customised yet satisfactory and effective smart footwear for monitoring diabetic neuropathy is complex. A statistical framework approach for evaluating smart insoles has been used [78], and it was found that computer-aided design and manufacturing (CAD-CAM) of SF achieves better offloading performance than the traditional shape-only-based approach. Hence, developing innovative tools to support the design and manufacturing of customised footwear for people with diabetes is a crucial step, and a CAD-based platform can help achieve this goal. Future research is needed to develop and optimize hardware tools and implement further design modules, namely insole–outsole, material selector, and valuator. However, the requirements of SF design may change according to a specific application or user group it is designed for, for example, Parkinson’s disease (PD), stroke, any injury, etc.

Aging can contribute to increased foot pain after prolonged daily activities. This may lead to foot supination (body weight falling on the outer edge of the feet) or overpronation (weight falling inwards). To avoid further complications caused by foot supination or overpronation, maintaining an appropriate posture while walking, standing, or carrying out any daily activities is important. Unaddressed, these foot overcorrections can otherwise worsen and may develop into foot disorders called gait disorders. Rehabilitation can effectively treat posture disorders, and biomechanics plays an important role in understanding posture [79]. In recent years, smart footwear has enabled the assessment of gait and mobility disorders using biomechanical parameters [79]. A study reported that a person’s gait pattern is strongly influenced by age, personality, and mood [80]. Foot impairments may affect daily activities and hence affect the quality of life. Several methods were proposed for developing smart footwear as an assistive device in cases of gait disorder [81,82,83,84,85,86]. Further, the material used in footwear directly affects users’ comfort, and a soft material-based smart insole could also provide equally good results [87]. Wu et al. [88] demonstrated that analysis of gait disorders can be successfully achieved in real time.

Gait analysis involves the evaluation of several parameters such as muscle strength related to limb activities, spatiotemporal joint kinematics, joint force, pressure distribution, plantar pressure, etc. Evidence exists for using an inertial sensor to detect events by measuring spatiotemporal parameters [89]. A similar approach can be found using multi-cell piezoresistive sensors, inertial measurement, and logic units for measuring stride length, velocity, and foot clearance employing the ML technique [90]. Various methods are reported for measuring CoP trajectories and Kinematic parameters [91], estimating the progression angle while walking [92], monitoring alcohol-impaired gait [93], measuring kinematic and kinetic parameters [94], and determining plantar pressure [95]. A smart insole PODOSmart system [96] measures spatiotemporal and kinematic gait parameters using wireless sensor technology and microprocessors.

Further, a pilot study was conducted to evaluate the performance of smart footwear with the Tread Port virtual reality system for providing gait training [97]. The study concluded that there is a requirement to boost the efficiency of smart footwear. Nowadays, custom-made capacitive sensors such as lightweight textile-based [98], Flexible Porous Graphene [99], and Polydimethylsiloxane composite material-based [100] can be found in smart footwear for monitoring posture by measuring foot pressure. Another study [101] tested the efficiency of textured and prefabricated insoles by inserting them in medical and sports shoes, obtaining a significant change in reach distance compared to going barefoot. In recent decades, the shift to more sedentary lifestyles is exacerbating muscle weakness and joint stiffness issues, increasing the risk of arthritis and osteoporosis due to lack of weight-bearing activities that strengthen bones. These are collectively referred to as musculoskeletal problems. Several researchers experimentally showed that SF can help mitigate musculoskeletal issues by providing support [100,102,103,104,105,106,107]. A detailed technology evolution addressing gait disorders can be found in a review article [108], covering various types of smart wearables and their benefits.

This section outlines the work of several research groups that addressed different patient monitoring applications by designing SF for specific applications. Technical insights and an overview of these approaches are provided in Table 3 for quick reference.

### 3.3. Application 3: Detection and Recognition (Classification of Disorders)

**Figure 9:**
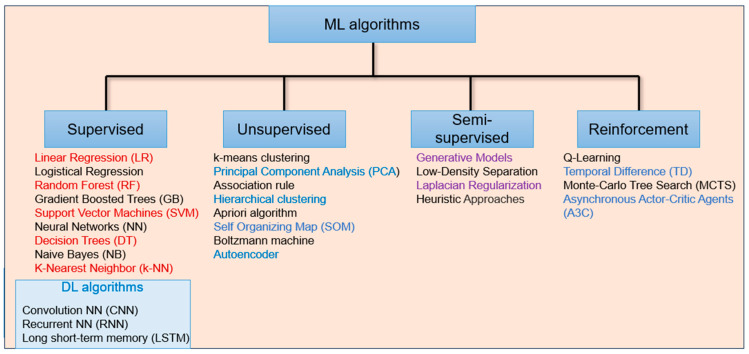
Different types of ML and DL algorithms.

Human data, such as physical and physiological parameters, can be captured using sensors for further analysis to aid decisions during diagnosis or treatment. Sensor data recorded via SF are typically time series, and extracting the required information using manual timestamping and analysis can be time consuming, resource intensive, and challenging. Hence, detection and analysis of sensor data recorded via SF for identifying underlying patterns to accurately categorise the type of disease is mainly performed automatically. ML and DL have recently been employed to automate sensor data analysis and detection. Different types of ML and DL algorithms used for healthcare applications are illustrated in Figure 9.

The commonly used algorithms in reported articles are highlighted in different colours for clarity. Using statistical methods, Jain et al. [109] classified accelerometer data for three walking activities. Another approach by Aqueveque et al. yielded promising gait analysis results by segmenting and analysing pressure data acquired from custom-made capacitive sensors made of two superimposed flexible copper films [110]. Classification of gait patterns was addressed using the traditional supervised ML approach [111,112,113,114,115,116] and the DL approaches [41,117,118,119,120]. Further, smart footwear data were used to train the NN to recognise foot pronation and supination [121]. A study by Moore et al. [122] compared the performances of several ML algorithms to predict the strike angle and classify the strike pattern, classifying the strike pattern and getting misclassification only in the case of the mid-foot strike pattern. A fall is an unintentional event leading to injury, and it can happen to normal people or people with gait disorders or during rehabilitation. Hence, many smart footwear designs can detect falls [123,124,125], reporting promising results for practical scenarios. Many researchers have concentrated on designing and developing smart footwear for gait analysis. However, the analysis result depends on the type of sensors used, data acquisition bandwidth, sampling, and visualisation. An experimental study can be found on the selection of bandwidth and sampling frequency for accurate classification of gait patterns [88]. An investigation by Codina et al. [126] demonstrated that wireless technology and mobile application integration into smart footwear could be used for gait analysis and recovery speed monitoring after hip surgery and can also be used for fall detection. Another method developed by Sudharshan et al. [127] employed a smartphone-based application using a decision tree algorithm to classify walking patterns, obtaining a classification accuracy of approximately 92%. The design included five pressure sensors, four vibrators, and a Bluetooth transmission. Walking pattern analysis and assistance are required for patients with Parkinson’s Disease (PD). A special footwear design employing closed-loop sensing to assist in the rehabilitation process of PD patients by analysing walking patterns was presented by Cai et al. [128]. In addition to the aforementioned smart footwear designs, a study showed the possibilities of smart socks to measure plantar pressure and analyse gait patterns [129]. The suitability of smart footwear for the recognition of many disorders and the identification of body parameters such as heart rate estimation [130,131], neuromuscular disease [132], postoperative outcome predictors [133], human behaviour classification using pneumatic actuators [134], and knee abduction moment prediction [135] have been investigated by researchers.

The present section provides details of SF data analysis for recognizing and classifying specific disorders. This was achieved either using statistical tools or by employing ML algorithms. Table 4 provides an overview of the methods that have reported disease or pattern classification. The table also highlights the outcomes of the approaches, enabling researchers to identify the gaps. A major issue that can be identified from the table is the smaller number of participants involved in the experiments, which limited the confidence and extrapolation of the results.

## 4. Observations and Future Perspectives

Smart footwear has been revolutionising the future of footwear with the introduction of technology in product design and development. From monitoring physical health attributes to evaluating health benefits, smart footwear can enable wearers to receive personalised feedback. Customised smart footwear equipped with sensors, controllers, and processors can assist and classify patterns. Based on the current survey, a few key observations have been made. The integration of sensors in smart footwear is continuously evolving, providing detailed health and fitness data for analysis. This includes tracking diverse health metrics, such as blood oxygen levels, heart rate, and energy expenditure. ML and especially DL algorithms enhance the accuracy and predictive capabilities of SFs, allowing for early detection of health issues based on changes in walking patterns or other metrics. Essentially, the ability of SF to become an efficient assistive device has been designed for individuals with disabilities, such as those who are visually impaired. Features like obstacle detection, GPS tracking, and machine learning-based object identification were used to help individuals navigate their environment more independently. The integration of wireless communication technologies also allows real-time alerts and updates to be sent to a user’s smartphone or other devices. The expansion of SF applications is also observed in healthcare applications, particularly for patients with diabetes and walking disorders. It has been demonstrated that footwear monitoring pressure, temperature, and humidity could help prevent and manage conditions like DFU. Further, optimising the sampling rate for data analysis has been reported to improve accuracy. The development of adaptive sampling algorithms was reported. This allows smart footwear to adjust its data collection based on the user’s activity level. Also, the use of smartphone apps to collect and analyse data from SF has been proposed in many studies. This will allow users to easily track their health and fitness progress, receive alerts and recommendations, and share data with their healthcare providers. Improved and user-friendly designs for long-term usage were also reported. However, battery life was the major concern in many intelligent SF designs. This was also addressed by employing energy harvesting and storing techniques based on walking, to power the footwear’s sensors and other electronic components. As shown in Figure 4, incorporating all these features increases the cost of the SF. However, cost-effectiveness is needed to make it accessible to a wide range of consumers. Although a lot of SF is available on the market, many studies in the literature highlight that the design and development of SF require further research attention. Important avenues for future SF research can be summarised as follows. As sensor technology continues to evolve, opportunities to develop new types of sensors that can monitor a broader range of health and fitness metrics increase. This could include sensors that can detect changes in blood flow, muscle activity, or other physiological parameters. Another aspect is energy harvesting and storage. There is significant potential for research into more efficient energy harvesting and storage methods. This could involve developing new materials or designs that capture more energy from walking and/or creating more efficient energy storage systems to power the footwear’s electronics for longer periods. More research is required on low-cost, energy-efficient, multi-parameter integrated sensor measurement units that can be used in SF. Clinically usable SF needs to include a decision support system for disease diagnosis. Hence, there is a need for ML and artificial intelligence (AI) methods to convert footwear to intelligent footwear. The use of these algorithms in SF is still relatively new, and there is much potential for further research in this area. This could involve developing algorithms that can more accurately predict health issues based on sensor data or creating AI systems that provide personalised advice. Also, there is a need for more research into how smart footwear can be used to assist individuals with disabilities. This could involve developing new features or technologies to help these individuals navigate their environment or studying how smart footwear can be integrated into existing assistive devices or systems. Further, designing SF that can prevent or manage specific medical conditions or developing new types of therapeutic footwear that can deliver targeted treatments is in demand. As SF becomes more common, research is needed to improve the user experience. This could involve studying how to make smart footwear more comfortable, user-friendly, and stylish and/or researching how to make the data collected by smart footwear more accessible and valuable to users. With the increasing amount of personal health data being collected by smart footwear, a question is how to protect this data. Hence, attention should be paid to developing new encryption methods and privacy protocols and studying how to educate users about the importance of data security. As devices become increasingly smart and interconnected, researchers can explore how smart footwear can be integrated with other devices, such as smartphones, smartwatches, or home automation systems. Ultimately, sustainability is an essential consideration in product design, and there would be research and innovation opportunities in developing smart footwear using sustainable materials and/or manufacturing processes. Consumers or end-users care about the cost of any such devices. Hence, this opens a new avenue for manufacturing cost-effective SF by understanding manufacturing techniques and production processes while maintaining quality and performance.

## 5. Conclusions

In conclusion, smart footwear represents a significant advancement in wearable technology, offering promising applications in health monitoring, assistive technology, and medical treatments. Integrating advanced sensors, energy harvesting systems, and machine learning algorithms can revolutionise personal health management and improve the quality of life for individuals with disabilities. However, further research is needed to overcome current challenges, including improving the comfort and design of smart footwear, reducing costs, and ensuring data privacy and security. Future research should also explore the integration of smart footwear with other smart devices, using sustainable materials and manufacturing processes, and the development of more efficient energy harvesting and storage systems. As the field of smart footwear continues to evolve, it holds a promise of transforming our understanding of personal health and fitness, and opening up new possibilities for assistive and therapeutic interventions.

## Figures and Tables

**Table 1:** Suitable sensors for SF design.

Sl. No	Sensor Type, Its Operation Principle, and Possible Applications in Smart Footwear
1	Ultrasonic sensors [3]: These sensors utilise ultrasonic waves to measure distance and detect objects. They are one of the most commonly used sensors in footwear, specifically for aiding people with visual disability. They can detect the presence or absence of objects within a specific range. Further, these sensors can measure insole thickness and footwear wear and tear and suggest replacement schedules.
2	LiDAR sensors [4]: Light detection and ranging (LiDAR) based Time of Flight (ToF) sensors are currently the preferred technology for automotive and drone applications. ToF sensors have the emitter, receiver, and processor system on the same PCB/package for easy, cost-effective, and small-footprint integration. They offer high-speed, precise distance measurement independent of target size, colour, and reflectance. A LiDAR sensor can be integrated into footwear to replace the traditional ultrasonic sensor or added as an additional sensor to support features such as pothole detection, obstacle warning, etc.
3	Pressure sensors [5]: Pressure sensors measure pressure by converting the applied pressure into an electrical signal that can be measured and utilised for various applications. Strain Gauge Pressure Sensors measure the strain or deformation caused by pressure. Capacitive Pressure Sensors use changes in capacitance. Changes in capacitance are used to measure pressure. Piezoelectric Pressure Sensors utilise the piezoelectric effect, where pressure generates an electric charge in certain materials. Resonant Pressure Sensors use change in the resonant frequency of a vibrating element under pressure to determine the applied force. They measure pressure at different points on the foot (placed in soles). Additionally, strain gauge sensors installed in the footwear can detect sudden bends and movements in the footwear. Pressure sensors can also be used to measure weight.
4	Accelerometers and Gyroscopes [6]: An accelerometer measures linear acceleration and can detect the movement of an object in terms of acceleration, deceleration, or changes in direction. On the other hand, a gyroscope measures angular velocity around a particular axis. It detects changes in orientation or rotational movements, such as tilting, rotating, or twisting. Inertial measurement units (IMUs) incorporate accelerometers and gyroscopes into a single sensor package, providing a more compact and integrated solution for motion-sensing applications. IMUs are integrated into wearable devices to monitor and analyse physical activities. They can measure steps, distance, speed, and calories burned and provide feedback on movement patterns and exercise techniques.
5	Sweat Sensors [3]: Skin-worn biosensors can analyse the wearer’s sweat to monitor various physiological conditions. Biomarkers in the sweat can be used to detect certain genetic conditions. Also, using the glucose-level correlation between sweat and blood leads to potential applications in the continuous monitoring of diabetes.
6	Temperature Sensors: Temperature sensors detect and measure the heat and coolness of air, liquids, or solid surfaces and convert them into electrical signals. Types of temperature sensor include: Resistance Temperature Detector (RTD) sensors, which measure temperature by changing resistance proportional to the temperature. These are available as individual sensors or fully packaged assemblies consisting of a sensor element, a covering, an epoxy or filler, extension leads, and sometimes connectors, thus allowing for flexibility in design [7]. Negative Temperature Coefficient (NTC) thermistors, which use the properties of ceramic/metal composites that have an inverse relation between resistance and temperature to measure temperature. NTC sensors have a small size, excellent long-term stability, high accuracy, and precision [8]. A thermocouple sensor is formed by joining two individually insulated dissimilar metals at one end. The temperature is measured at this joint. When the joint is placed in a high-temperature environment, a small voltage is produced at the open ends of the two metals, which can be measured and interpreted [9]. Thermopile infrared (IR) temperature sensors, which generally provide non-contact temperature measurements. These are composed of small thermocouples on a silicon chip that absorb the incident IR energy and produce an equivalent output signal. Modern sensors also have an integrated reference sensor for calibration and compensation. The output can be an analogue voltage or a digital value [10]. Digital Temperature Sensors, which are IC temperature measurement systems. The miniature packages are designed specifically for tight spaces. The integrated microcircuit design allows quick response to changes in process temperature, fast conversion times, and very low power consumption [11]. Temperature sensors can be used in smart footwear to keep track of foot temperature and act as surrogate activity markers. Temperature changes in specific foot areas can indicate inflammation, injury, or conditions like diabetic foot ulcers. Elevated temperatures in localised regions might signify inflammation or infection. They can also assist in monitoring blood circulation in the feet. Changes in foot temperature might indicate poor circulation.
7	Gas sensors can be used to detect foot odour, and they can detect Bromodosis, possibly caused by fungal infection [12]. Bromodosis is smelly feet, and it is often caused by the interaction of sweat with bacteria on the skin’s surface. Fungal infections like athlete’s foot or other dermatophyte infections can also contribute to foot odour. Gas sensors can detect the specific gases emitted by these fungi, aiding in the early identification of such infections.

**Table 2:** Details of smart footwear available in the market [14].

Smart Footwear (Name of the Company)	Applications	Type of Sensor (No. of Sensors Used)	Pressure (kPa)	IE	MDAR (Hz)	DTT	BA (Hrs.)	Cost
WIISEL	Continuous gait monitoring, analysis & fall risk assessment	Piezoresistive (14)	350	Yes	33.3	BLE	16	—
Pedar (Novel)	Footwear design and injury prevention	Pressure (99)	600	No	100	BT	1	15,540 €
F-Scan (Tekscan)	Gait analysis & biomechanics, diabetic offloading, sports medicine	Pressure (960)	862	No	165	USB, Wi-fi	0.2	16,000 $
BioFoot (IBV)	Sports gait analysis, footwear design	Pressure (64)	1200	No	500	Wi-Fi	1	12,995 €
paroLogg /parotec (paromed)	Foot pressure analysis	Pressure (32), Inertial	625	No	300	Wi-fi	1.5	—
Foot Pres- sure MS (Medilogic)	Gait, sports, health prevention, prosthesis and orthotics, diabetics	Solid State Relay (SSR) sensors (240)	640	No	300	Wireless	—	—
Smart Step	Rehabilitation process	—	—	No	—	Card	—	6000 $
Smart Insoles (24 eight, LLC)	Medical, sports, and gaming	Pressure (4), Inertial	241	Yes	—	Wireless	100	—
OpenGo science (Moticon)	Medical & sports science, Rehabilitation & training analysis	Pressure (13), Inertial	400	Yes	100	Wireless	—	2000 $
Footswitches Insole (B & L Engineering)	Gait analysis	Pressure sensors (4)	—	No	—	Wireless	—	9000 $

IE—Integrated Electronics, MDAR—Maximum Data Acquisition Rate, DTT—Data Transfer Type, BA—Battery Autonomy.

**Table 3:** Technical insights and overview of SF design for patient monitoring.

Ref. No.	Target Application	Technical Details	Main Findings
[47]	Inertial and plan- tar pressure measurement	Insole, wrist band, accelerometer, gyroscope, pressure sensor, BLE, smartphone, sampling rate 50 Hz.	The best body part for HAR: Feet or wrists.
[48]	Six ambulation activities detection	Smart insoles: accelerometer, gyroscope, magnetometer, ECU, BLE, ML algorithms, smartphone, 200 Hz, 120 min, 25–55 years.	Inertial sensors are reliable for dynamic and pressure sensors for stationary activities.
[53]	Foot pressure distribution	Capacitive sensor, ML.	ML provides the required pressure measurement.
[50]	Plantar pressure and activity recognition	Seven pressure sensors, FFT, ML, 100 Hz, 12-bit, 26 ± 9 years.	Generalization is needed for larger populations.
[54]	Foot pressure and motion activities	280 capacitive pressure sensors, 56 temperature sensors, FT, wired.	Smart insole alternative for activity recognition.
[55]	Plantar pressure – daily activity	MWCNTs/PDMS piezoresistive nanocomposites, LAB View.	Useful for disease detection and diagnosis.
[56]	Daily activities recognition	Accelerometer, DL, wireless.	SF is user-friendly for all ages.
[58]	Locomotor activities	Accelerometer, gyroscope, magnetometer, FFT, CNN.	User-independent system for HAR possible.
[62]	Diabetic feet monitoring	Temperature, humidity sensors, eight pressure sensors, BLE, Arduino 328, 25–55 years.	Improves self-management and health outcomes.
[64]	DFU prevention	Flexible insoles, 99 capacitance-based sensors, 50 Hz, 2 sensors/cm^2^, 919 patient’s databases.	Pre-clinical studies met user needs.
[67]	DFU monitoring: plantar pressure	Eight pressure sensors, a smartwatch, 8 Hz, and an age group greater than 18 years.	Continuous monitoring reduces DFU recurrence.
[68]	DFU: Plantar pressure measurement	Eight capacitive sensors, flexible PCB, BLE, microcontroller, 100 Hz, 28 bits.	Enhances efficiency in studying diabetic foot conditions.
[70]	DFU monitoring: Plantar pressure	Pressure sensor, PC, 50 Hz, 76 participants.	Optimization is needed for real-time use.
[71]	Diabetic foot monitoring	Four temperature sensors, 35 participants.	Continuous monitoring provides preventative foot ulcer information.
[73]	DFU: pressure measurement	Nineteen female participants, 57–75 years, 4D scanner.	Custom insoles and heel pads help redistribute pressure.
[74]	DFU monitoring: Temperature, humidity	Textile insole, silicon tubes, leather, five sensors, and 21–30 years of age females.	Textile insoles enhance thermal comfort.
[81]	Balance and gait analysis in older women	Thirty women, 65–83 years, lab tests, ethyl vinyl acetate insoles.	Significant reduction in step width observed.
[82]	Gait analysis and PD study	Pressure sensors, accelerometer, 29 participants, 100 Hz, 20–59 years.	Dataset valuable for detailed gait analysis.
[83]	Fall detection in elderly	Arduino Nano, sensors array, buzzer, vibration motor.	Smart shoes with devices detect and prevent falls.
[84]	Mobility and gait assessment	Force sensing resistors, IMU, ultrasound sensor, Arduino, BLE.	Detects abnormalities in walking patterns.
[86]	Flat feet detection	Three force sensors, accelerometer, BLE, Arduino Nano.	Cost-effective alternative to motion capture systems.
[87]	Real-time gait monitoring	Soft insole, capacitance-based pressure sensor, conductive textile, microcontroller, 100 Hz, 15 participants.	Textile-based insole alternative to smart shoes.
[90]	Portable gait analysis	Piezoresistive sensor, IMU, logic unit, 500 Hz, 6 min recording, ML, 14 participants.	Learning-based methods improve gait parameters.
[91]	Gait parameters measurement	Piezoresistive sensors, microcontroller, WIFI, IMU, 500 Hz, 9 participants, MAT- LAB.	Useful for out-of-lab gait analysis.
[92]	Foot progression angle estimation	Inertial, magnetometer units, accelerometer, gyroscope, 100 Hz, 14 participants, 22–29 years.	Useful for knee osteoarthritis monitoring in daily life.
[93]	Detecting changes in gait by alcohol intoxication	Twenty participants, wireless mode, ML algorithm.	SF can be used for detecting alcohol-impaired gait.
[94]	Locomotion monitoring: real-time kinetic measurement	Pressure sensors, IMUs, WIFI, Smartphone, PC, sampling rate: 100 Hz, 9 participants, MATLAB 2019b software.	Acceptable matches were achieved for the measured CoPx and the calculated knee joint torques out of 13 movements.
[95]	Plantar pressure measurement	Capacitive sensor: silver and cotton, microchip, USB, laptop, BLE.	Gait phases and different patterns can be detected, and the system is bacterial-resistant.
[96]	Gait analysis tool: PODOS-mart^®^	IMUs: Sensors, 11 participants, age group: 20–49 years, BLE, sampling rate: 208 Hz.	Ease of use without technical education.
[97]	Evaluating haptic terrain for older adults and PD patients (TreadPort)	Five bladders, PC, VR terrain, WIFI, microcontroller, CAVE display, camera: 60 frames/sec.	Applicable for gait training for walking impediments caused by PD.
[98]	Locomotion monitoring: centre of pressure detection	Five textile capacitive sensors, WIFI, sampling rate: 100 Hz, MATLAB R2021a software.	Smart wearable sensors can improve quality of life.
[99]	Designing and fabricating biomimetic porous graphene flexible sensor: gait analysis	Graphene nanoplates, SBR foam, silver electrodes, microcontroller, BLE.	The system can monitor older and can help with gait training.
[100]	Plantar pressure measurement: gait analysis	Twelve capacitive sensors: copper and poly-dimethyl siloxane, PIC microcontroller, BLE, PC.	The design offers correct performance behaviour under footfall.

**Table 4:** Overview of SF data analysis for disease recognition and classification.

Ref. No.	Study Objectives	Techniques	Target Group	Outcomes
[109]	Stride segmentation of accelerometer data, classification of three walking patterns.	TinyML, edge computing.	-	Mean stride duration is around 1.1 with a 95% confidence interval.
[110]	Gait segmentation method based on plantar pressure only.	Thresholding: moving average, statistical analysis.	Six participants: 19–29 years.	The calculated distribution between stance-phase and swing-phase time is almost 60%/40%—aligned with literature studies.
[111]	Gait classification using feature analysis.	ML algorithms: RF, k-NN, LR, SVM.	Eighteen participants: 22–31 years.	A combination of accelerometer and gyroscope sensor features with SVM achieved the best performance with 89.36% accuracy.
[112]	Gait pattern classification.	ML algorithm: NN.	Eleven participants: 22–33 years.	A built-in accelerometer and gyro sensor gait-pattern classification system can be used without the constraints of a controlled environment.
[113]	Detecting 13 commonly used human movements.	ML algorithms: PCA, k-NN, ANN, SVM.	Thirty-four participants: average age 22.6 years.	The model proved to be effective, with an accuracy of 86%.
[114]	Gait pattern classification.	ML algorithm: NN.	Six participants.	The architecture with three nodes provided effectiveness metrics above 99.6%.
[115]	Gait pattern classification.	ML algorithms: k-NN, SVM, ELM, MLP.	-	ELM performed better, with an overall accuracy of 93.54%.
[41]	Detecting walking behaviour.	ML algorithms: NN, DL (CNN).	Three participants: 26–27 years.	The best performance was achieved with convolutional layered ANN with an average accuracy of 84%.
[117]	Gait type classification.	DL (CNN)	Fourteen participants: 20–30 years.	Experimental results for seven types of gait showed a high classification rate of more than 90%.
[118]	Gait abnormality detection.	DL (CNN)	Twenty-one participants: 24–37 years.	Deployment of CNN-LSTM in Nordic nRF52840 can be revisited with model- pruning and post-training quantization.
[119]	Walking pattern analysis.	DL	Video frames.	SF can detect any injury the shoe user is suffering from.
[120]	Abnormal gait pattern recognition.	DL (LSTM- CNN)	Twenty-five participants: avg. age 22 years.	A personalised gait classification approach, which is accurate and reliable.
[121]	Recognition of foot pronation and supination.	DL (NN)	Six participants.	The system can adequately detect the three footprints’ types with a global error of less than 0.86.
[122]	Foot strike pattern classification.	ML algorithms: LR, conditional inference tree, RF.	Thirty participants: 27–41 years.	The system aided in the research and coaching of running movements & obtained the highest classification accuracy of 94% using RF.
[124]	Fall detection.	Statistical tool and algorithm.	Seventeen participants: 21–55 years.	The method demonstrated satisfactory performances providing a maximum accuracy of 97.1%.
[125]	Fall detection.	Advanced Fall detection algorithm.	Six participants.	The insole can measure walking speed, the distance covered, and the measurement of balance or weight.
[88]	Gait analysis and monitoring.	PCA, event detection algorithm.	Four participants.	The new gait metric (eigen analysis) has great potential to be used as a powerful analytical tool for gait disorder diagnostics.
[127]	Identification and correction for people with abnormal walking patterns.	ML algorithm: DT.	One thousand two hundred fifty data points—five classes with 250 data points.	The machine learning approach has a 91.68% accuracy and shows promise for assisting people with arthritis.
[129]	Gait analysis.	Sum of Manhattan distances (SMD).	Three participants.	Smart socks can be an alternative to smart shoes.
[130]	Heart rate estimation.	DL (LSTM- CNN)	Fifteen participants.	Significant levels of heart rate estimation could be made using SF with a correlation of 0.91.
[131]	Heart rate and Energy expenditure estimation.	DL (CNN)	Ten participants: 20–24 years.	Estimations can be accurate by effectively selecting the optimal sensors.
[133]	Gait analysis.	Multivariate analysis, statistical tool.	Twenty-nine participants: 43–75 years.	SF is ideally suited for preoperative evaluation in the clinical setting.
[134]	Human be- haviour classification.	ML algorithm: RF.	Six participants: 20–22 years.	Four types of behaviour were classified with an F-measure of 78.6%.
[135]	Knee abduction movement detection.	ML algorithm: MLP regressor.	One participant: 24 years.	The system performed well in predicting KAM with an accuracy of 87%. However, more experimentation is required.

## Data Availability

Not applicable.
